# Formation and manipulation of cell spheroids using a density adjusted PEG/DEX aqueous two phase system

**DOI:** 10.1038/srep11891

**Published:** 2015-07-06

**Authors:** Chungmin Han, Shuichi Takayama, Jaesung Park

**Affiliations:** 1 Department of Mechanical Engineering, Pohang University of Science and Technology, Pohang, Gyeong-buk, Republic of Korea; 2 Department of Biomedical Engineering, Department of Macromolecular Science and Engineering, University of Michigan, Ann Arbor, MI, USA; 3 School of Interdisciplinary Bioscience and Bioengineering, Pohang University of Science and Technology, Pohang, Gyeong-buk, Republic of Korea

## Abstract

Various spheroid formation techniques have been widely developed for efficient and reliable 3-D cell culture research. Although those efforts improved many aspects of spheroid generation, the procedures became complex and also required unusual laboratory equipment. Many recent techniques still involve laborious pipetting steps for spheroid manipulation such as collection, distribution and reseeding. In this report, we used a density-controlled polyethylene glycol and dextran aqueous two phase system to generate spheroids that are both consistent in size and precisely size-controllable. Moreover, by adding a few drops of fresh medium to the wells the contain spheroids, they can be simply settled and attached to the culture surface due to reduced densities of the phases. This unique attribute of the technique significantly reduces the numerous pipetting steps of spheroid manipulation to a single pipetting; therefore, the errors from those steps are eliminated and the reliability and efficiency of a research can be maximized.

Most cells in tissues and organs form three dimensional (3-D) structures which facilitate physiological functions by enabling close interaction of cells with other cells or with the extracellular matrix^1^,^2^. However, traditional 2-dimensional cell culture systems have not been able to replicate these biological characteristics because intercellular interactions among cells on flat plates are different from those in *in vivo* tissues^3^. To overcome this limitation, various types of 3-D culture methods have been developed that use such techniques as filter inserts, polymer scaffolds, hydrogels, and microfluidic chips^1^,^4^,^5^,^6^,^7^. Among those methods, spheroids or cell-aggregate culture methods are technically simple, and mimic tissues’ characteristics well, so these methods have been most widely utilized for practical applications such as drug development and stem cell differentiation^8^,^9^.

Various techniques such as hanging drops, spinner flasks, non-adherent surfaces and micro-fabricated scaffolds have been developed for efficient and reliable generation of spheroids^10^,^11^. Recent techniques such as microfluidic chips, stimulus-responsive hydrogels and magnetic levitation achieved better efficiency and the easier spheroid manipulations than the earlier techniques^12^,^13^,^14^. Although these new approaches have improved many aspects of spheroid formation, they require complex procedures and unusual materials such as magnetic levitation equipment and microfabrication equipment, and entail tedious pipetting steps to manipulate spheroids for further analyses and applications. High-throughput spheroid formation systems were also developed to alleviate those problems, but the systems are generally less suitable for single spheroid analyses than are the existing techniques^15^.

Aqueous two-phase systems (ATPSs) that use polyethylene glycol (PEG) and dextran (DEX) have been introduced to generate two-dimensional patterns for advanced cell cultures^16^. Phases of these ATPSs have different physical and chemical properties, and therefore have different affinities to cells and biomolecules, so cells can be unequally partitioned and patterned only in one of the phases^17^. This ATPS patterning method is simple and does not require special laboratory equipment such as microfabrication tools, so it has widely been used in various studies such as stem cell-feeder cell interactions and bacterial chemical communication studies^18^,^19^. However, these studies mainly focused on 2-D cell patterning but not on 3-D cell culture because they overlooked the physical properties of phases such as density that can float the cells.

In this study, we developed a new spheroid generation method that uses density-adjusted PEG/DEX ATPS patterns, and which is compatible with various types of cell that aggregate into spheroids. This new method mainly exploits the relative densities of DEX-rich phase and spheroid-forming cells; when cells in DEX-rich pattern are less dense than the DEX-rich phase, they float and gather at the apex of the DEX-rich pattern in PEG. These gathered cells form a spheroid when the interaction between them is strong enough. The spheroids formed using ATPS could be transferred and maintained in conventional suspension culture formats for further uses. In addition, the spheroids can also be released from the DEX-rich phase and patterned on a culture plate simply by adding a few drops of PEG/DEX-free fresh medium, which changes the density of the phases to be less than that of the cells. This method can simply switch culture mode from a floating to adhesion culture without changing culture vessels or transferring spheroids, and can simplify procedures of spheroid research. We demonstrated this method successfully for a study of embryoid body (EB) formation and differentiation, in which both floating spheroid culture and adhesion culture methods are commonly used.

## Results

### ATPSs and formation of DEX-in-PEG ATPS pattern

**Figure 1:**
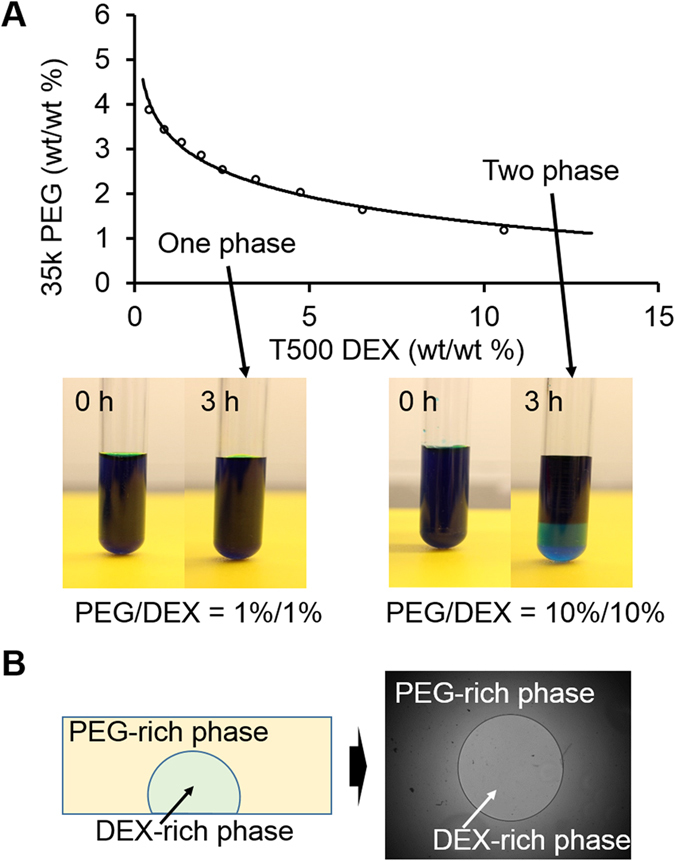
ATPSs consist of 35 K PEG and 500K DEX. (**A**) Experimentally-acquired phase diagram of 35 K PEG and 500 K DEX in PBS. Points above the curve can form two immiscible liquid phases whereas points below the curve cannot. The bottom phase is DEX-rich; the top phase is PEG-rich phase. Formation of the two phases can be easily visualized using colored food dye which is preferentially partitioned to one phase (here, blue food dye partitioned to the PEG-rich phase). (**B**) DEX-in-PEG ATPS pattern. Due to its immiscible nature, DEX-rich phase can be patterned inside the PEG-rich phase. Successful pattern showed a clear circular PEG-DEX interface and was stable for ~5 d.

Based on the phase separation diagram, we selected eight different PEG/DEX ATPSs that had DEX concentrations that were all in the two-phase-forming region (Fig. 1A). The formation of two phases was checked using blue food-dye which is preferentially partitioning to PEG-rich phase when PEG/DEX ATPS is formed (Fig. 1A). The top (PEG-rich) and bottom (DEX-rich) phases were then separated and transferred to new containers and cleaned by following centrifugation. The two prepared phases were patterned as DEX drops in PEG reservoirs in a 96-well plate (Fig. 1B). A successfully-formed DEX-in-PEG ATPS pattern showed a clear circular boundary between the phases under a phase-contrast microscope, and remained stable and immiscible for >5 d until the medium had substantially evaporated (Fig. 1B).

### Spheroid formation using DEX-in-PEG ATPS pattern

**Figure 2:**
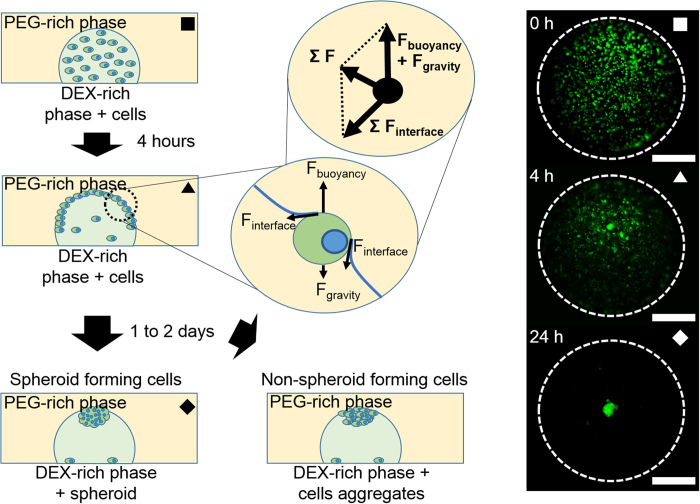
Spheroid formation using DEX-in-PEG ATPS pattern. (**A**) ATPS spheroid formation. When cells were co-patterned with DEX-rich phase in PEG-rich phase, cells were confined inside DEX droplets as a uniform cell suspension (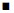). Four hours later, most of the cells had risen due to buoyancy force and had become trapped by interfacial surface tension at the interface of PEG and DEX (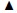). In this stage, net force in the tangential direction of the meniscus was exerted on the cells. Therefore cells were forced to move toward the apex of the DEX droplet, where the net force became zero. One to two days after patterning, cells gathered at apex attached tightly to each other and formed a spheroid. (♦) Non-spheroid forming cells formed a loose cell aggregate because cell-cell adhesion was not sufficient. Dotted lines: interfaces of DEX drops in PEG reservoirs. Scale bar: 500 μm.

When the DEX-in-PEG pattern was formed with DEX-rich phase containing cells, the cells were initially confined to the DEX-rich phase as a homogeneous cell suspension (Fig. 2 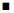). About 4 h later, most of the cells had floated from the DEX-rich phase and finally became trapped at the phase interface along the DEX drop meniscus (Fig. 2 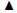). In this state, several forces act on the trapped cells, mainly surface tension force from the interface and buoyancy force from the density difference. Because the interaction between cells and DEX-rich phase is more favorable than the interaction between cells and PEG-rich phase, a contact angle between cells and two phases forms (Fig. 2, magnified schematics, Fig. S1). This contact angle formation exerts surface forces in a tangential direction to the contact point; therefore the main forces can be simplified as a free body diagram (Fig. 2). As a result of this force interaction, a cell trapped at the interface moves along the phase interface until it reaches the apex of the DEX drop meniscus, where the forces are balanced. One to two days after pattern formation, most of the cells had gathered at the apex of the DEX drop and formed a tight cell spheroid or loose cell aggregate depending on cell characteristics (Fig. 2 ♦). Fluorescence microscopic pictures (Fig. 2, right) were captured for CMFDA-labeled NIH-3T3 spheroids using DEX-in-PEG ATPS pattern.

### Density dependency of spheroid formation in various concentration ATPSs

**Figure 3:**
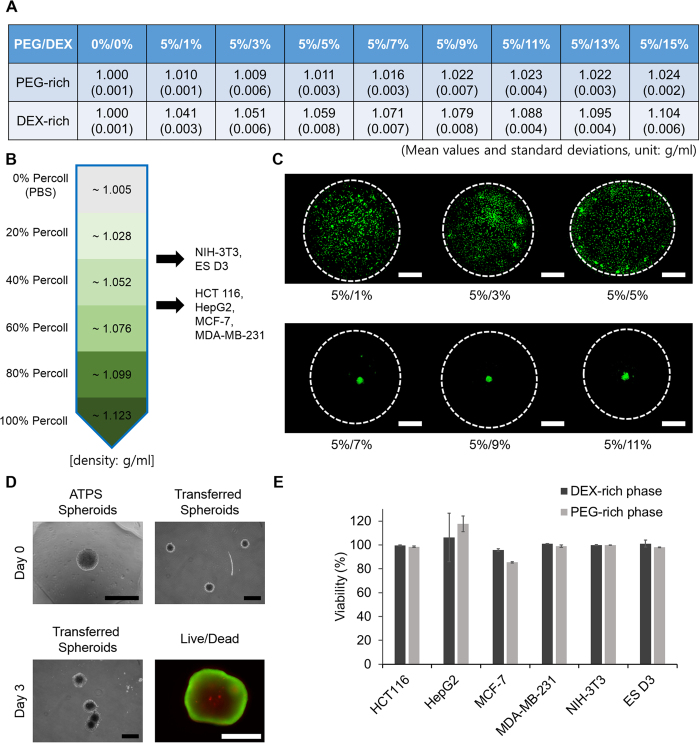
Density dependency of spheroid formation using DEX-in-PEG ATPS pattern. (**A**) Mean and standard deviation (n = 3) of densities of PEG- and DEX-rich phases in various concentration ATPSs. As DEX concentration increased, the density of DEX-rich phase increased substantially but the density of the PEG-rich phase increased only slightly. (**B**) Densities of cell lines used in spheroid formation by PEG/DEX ATPS pattern. Most of the cells were found between 60 to 40% and 40 to 20% Percoll layers. The densities of the Percoll layers were calculated following the manufacturer’s manual. (**C**) Cell tracker (CMFDA) labeled NIH-3T3 cells were tested for spheroid formation with 6 different PEG/DEX ATPS patterns which covers all the density of 20 to 60% Percoll layers. When the density of DEX-rich phase was less than or similar to the density of the cells, they settled and attached to the culture surface (PEG/DEX 5%/1% to 5%/5%). However, when the density of DEX-rich phase exceeded the density of the cells, they floated, gathered and formed a spheroid at the apex of the DEX-rich pattern (PEG/DEX 5%/7% to 5%/11%). Dotted lines: PEG/DEX interfaces. Scale bar: 500 μm. (**D**) Stability of spheroids formed using ATPS. The spheroids formed using ATPS were carefully transferred to 30 mm glass-bottom dish. After 3 days, spheroids were generally grown and some of them were merged. The spheroids were then stained with calcein AM and ethidium homo-dimer solution for live/dead analysis. Scale bar: 400 μm for phase-contrast images, 200 μm for fluorescence image (**E**) The viability of cells cultured with PEG- and DEX-rich phases for 48 h. ATPS (5%/9%) showed almost no cytotoxic effect.

To confirm the importance of the buoyancy force in this method of generating spheroids, densities of PEG- and DEX-rich phases of different ATPSs were measured (Fig. 3A). The densities of cells used in spheroid formation experiments were also measured using Percoll gradient centrifugation to compare with the densities of phases. Most of the cell types were detected between the 20% and 60% Percoll layers which have a density range from ~1.028 to ~1.076 g/ml (Fig. 3B). Because this density range includes the densities of DEX-rich phases from (wt% PEG/wt% DEX) 5/1, 5/3, 5/5, 5/7, 5/9 and 5/11 ATPSs, spheroid formation of NIH-3T3 was tested with patterns formed using those six DEX-in-PEG ATPSs. In the low-density ATPS patterns (5/1, 5/3 and 5/5) the NIH-3T3 cells settled and attached to the plate surface (Fig. 3C) because of insufficient buoyancy. The 5/7 ATPS pattern has higher density than the cells, so many of the cells started to gather at the center (apex) of the pattern and formed a spheroid. In the 5/9 and 5/11 ATPS patterns a large number of cells gathered and formed spheroids. When the PEG/DEX concentration was >5/11, cells formed dark and irregular cell aggregates, not spheroids (data not shown). To evaluate possible cytotoxicity of ATPS, cells were cultured in 5/9 ATPS, and their viability was compared to that of cells cultured in full growth media. The cells cultured in ATPS generally showed ~95% of that observed in full growth media (except MCF-7 cells cultured in PEG-rich phase (85% viability)) (Fig. 3E, Fig. S2). To test the stability of spheroids, spheroids formed using ATPS were carefully transferred using a 200-μl pipette to a 30-mm glass-bottom dish filled with normal growth medium (Fig. 3D). After 3 days of culture, transferred spheroids had generally grown and some had merged. The viability of the cells within the spheroids was confirmed by live/dead staining (Fig. 3D).

### Spheroid formation compatibility test with various cell types

**Figure 4:**
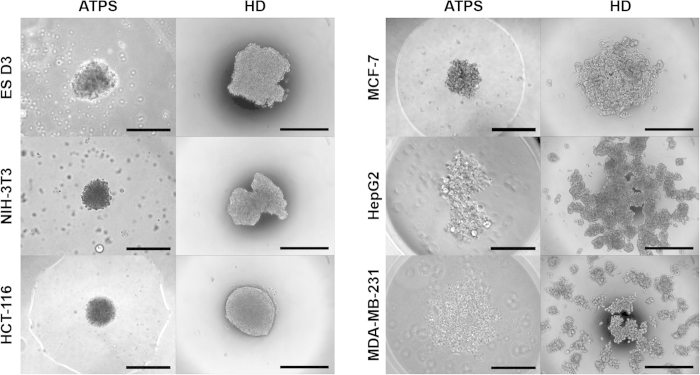
Spheroids of various cell lines formed using DEX-in-PEG ATPS and HD method. Well-known spheroid forming cells (ES D3, NIH-3T3, MCF-7 and HCT 116) showed regular-shaped and tight cell spheroids 48 h after ATPS patterning. Non-spheroid forming cell (MDA-MB-231) and slow-spheroid forming cell (HepG2) cell showed loose cell aggregates and irregularly-shaped aggregates respectively. Cells cultured with HD method showed similar but less effective spheroid forming capacity in terms of sphericity and tightness. Scale bars: 400 μm.

To investigate the compatibility of our ATPS spheroid formation, we assessed the abilities of six different cell types to form spheroids. Because almost all types of cells will eventually form cell aggregates after long intervals, we limited the spheroid formation time to 48 h to observe the differences in spheroid-making capability among cell types. In addition, excessive exposure of cells to dextran may cause vacuole formation and dextran internalization, and these processes may cause cytotoxicity^20^. Cells that are known to form spheroids (ES D3, NIH 3T3, MCF-7 and HCT 116) formed relatively compact and regular cell spheroids; a cell line that does not form spheroids (MDA-MB-231) was not able to form compact aggregates within 48 h (Fig. 4)^21^. HepG2 is also known to form spheroids but it also did not form spheroids within 48 h (Fig. 4); instead it formed dark and irregular cell aggregates after 96 h (data not shown). To assess the morphological characteristic of ATPS spheroids, the same sets of cells were applied to conventional hanging drop (HD) spheroid formation for the same period of time. After 48 h, spheroids formed using ATPS were more spherical and tighter than were the spheroids formed using HD (Fig. 4). HepG2 and MDA-MB-231 cells were not able to form spheroids within 48 h with HD method (Fig. 4). Therefore the ATPS spheroid formation method may be widely compatible with cells that form spheroids quickly, including very sensitive ES cells, but may not be compatible with cells that form spheroids slowly.

### Size consistency and size controllability of ATPS spheroid formation

**Figure 5:**
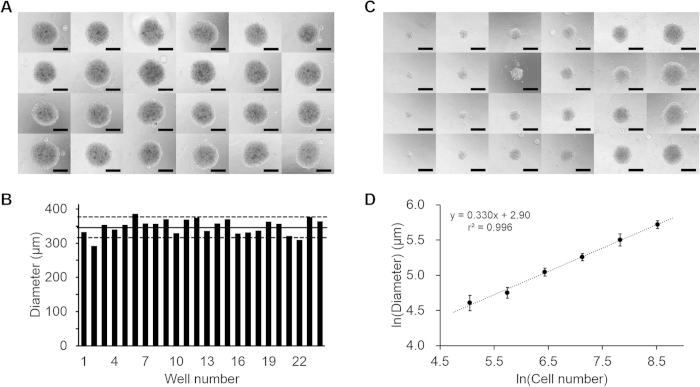
Size consistency and size controllability of spheroid formation using DEX-in-PEG ATPS pattern. (**A**) Twenty-four spheroids of ~5,000 HCT 116 cells from 24 DEX-in-PEG ATPS pattern in 96-well plate. The size and shape of the spheroids seemed consistent under a phase contrast microscope. Scale bar: 200 μm. (**B**) Measured Feret’s diameters of 24 HCT 116 spheroids. Spheroids had average diameter 347.9 μm (solid horizontal line) with standard deviation 23.0 μm (dotted lines, mean ± one standard deviation); 20 of the 24 spheroids were included in this range. (**C**) Phase contrast microscope images of four spheroids for each group (5000, 2500, 1250, 625, 312 and 156 HCT 116 cell spheroids) were generated using DEX-in-PEG ATPS patterns in a 96-well plate. Size increased cell number increased. Scale bar: 200 μm. (**D**) Feret’s diameter (y) vs. number of cells (x). Regression, ln(y) = 0.330∙ln(x) + 2.90, r^2^ = 0.996 (bars: ±1 sd, n = 6).

Because the consistency of spheroid formation greatly affects the repeatability of experimental results, the shapes and sizes of ATPS formed spheroids were observed using an inverted microscope. The spheroids for this consistency test were formed using 5,000 HCT 116 cells in 0.5 μl DEX drops patterned inside of PEG in a 96 well plate. The ATPS method successfully induced formation of a tightly-packed spheroid in each of the 24 wells (Fig. 5A). The mean Feret’s diameter was 347.9 μm (standard deviation sd = 23.0 μm) (Fig. 5B). The sd was <10% of the mean value, and ~80% of the spheroids (20/24) had diameters within one sd of the mean; these results indicate that the ATPS method induces spheroids with consistent size.

Size controllability of spheroid formation methods is also crucial because the size of the spheroid can be a key factor in spheroid research. In a size-controllability test, spheroids were formed using 156 to 5000 cells HCT 116 cells per DEX drop. Spheroid sizes increased as cell number increased, and size variations among spheroids with the same cell number samples seemed small (Fig. 5C). These images were also then processed to quantify the diameter of spheroids. The diameter of a sphere is proportional to the cube root of its volume (here approximated as cell number), so the logarithms of diameter and cell number should show a linear relationship with a slope of 1/3. The slope of the regression of ln(diameter) on ln(cell number) was 0.330 (Fig. 5D); thus the aggregates were spherical. This is evidence that the ATPS method can efficiently form various sizes of cell aggregates of almost spherical shape without substantial cell loss.

### Spheroid release and attachment to surface

**Figure 6:**
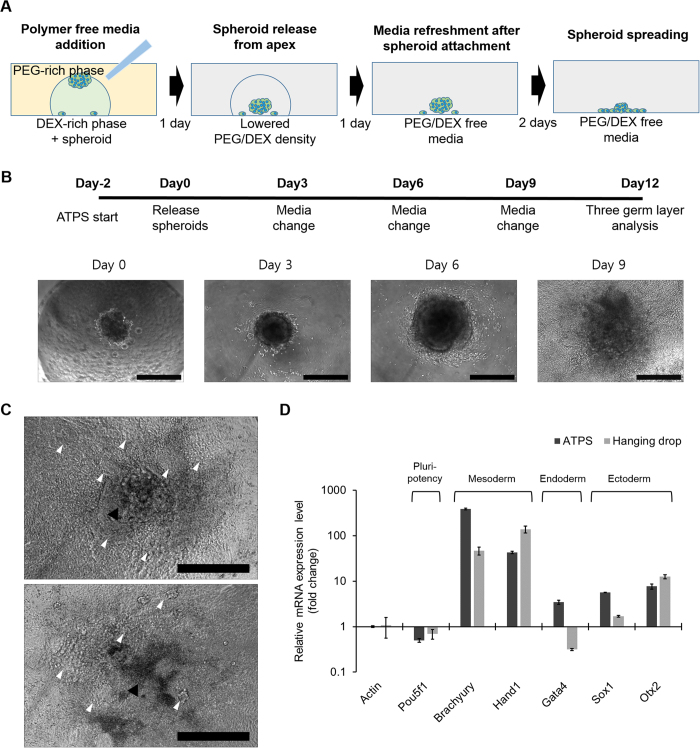
Spheroid release and culture mode switching from floating to adhesion culture. (**A**) Adding PEG/DEX-free fresh medium decreases the density of the DEX-in-PEG ATPS pattern; therefore floating spheroids settled. (**B**) EB formation and cardiac differentiation using DEX-in-PEG ATPS pattern. Ten EBs were formed with 5000 ES D3 cells using DEX-in-PEG ATPS pattern made of ES growth media in 96-well plates. (Day -2)Two days after pattern formation, formed EBs were released from the pattern apex and attached to the culture surface. (Day 0) 24 h later, EB attachment to the culture surface was confirmed using a microscope, and media were replaced with cardiac differentiation media. (Day 1) On day 9, beating clusters were observed in some of the EB cultures; on day 12, the number of beating clusters was counted under microscope. Scale bar: 400 μm (**C**) Representative images of day 12 EBs. Black arrow heads: EB cores; white heads: beating clusters which have different tissue morphology. Scale bar: 400 μm (**D**) qPCR analysis of representative three germ layer lineage markers. Relative mRNA expression levels of one pluripotency (Pou5f1), two mesodermal lineage (Brachyury and Hand1), one endodermal lineage (Gata4) and two ectodermal lineage markers (Sox1 and Otx2) were measured. The expression levels of all the markers were normalized by actin expression and then further divided by the normalized expression levels of each marker from undifferentiated ES cells. Gene expression of spheroids formed using HD method were also analyzed to be compared with the results of ATPS method (n = 3).

Because the formation of spheroids in the ATPS method is mainly affected by the density of phases, the culture mode can be easily switched from a floating spheroid culture to a surface-attached adhesion culture by reducing the density of ATPS. Addition of 200 μl PEG/DEX free medium to 100 μl ATPS patterns in a 96-well plate reduced the density of the culture medium to about 33% of the that of the original medium, so the spheroids became negatively buoyant and therefore settled to the bottom of the culture vessel. These spheroids were further cultured for 24 h to form cell-surface adhesions. For analysis, ATPS media was removed, the culture was washed twice with PBS and new fresh medium was added, and the spheroid-surface adhesion was observed under a microscope (Fig. 6A). Alternatively, by generating and releasing a spheroid on a previously-formed cell monolayer, a spheroid-monolayer co-culture can also be simply prepared. To further demonstrate this, NIH-3T3 cells that had been labeled with green fluorescent dye (CMFDA) were patterned with DEX-in-PEG ATPS above the pre-formed and unlabeled NIH-3T3 monolayer and released to form a spheroid-monolayer co-culture (Fig. S3).

### Embryoid body formation and differentiation

To demonstrate the research applicability of the ATPS method, we performed an experiment to examine embryoid body formation and differentiation in 96-well plates. Initially, ten EBs were generated using 5000 ES D3 cells with a 5%/9% PEG/DEX ATPS pattern made of ES cell growth medium, then attached to the surface by adding fresh medium (Fig. 6B). After attachment, spheroids were washed twice with PBS and cultured in ES cardiac differentiation medium for 12 d. At day 12, eight out of ten EBs had successfully attached and had spread on the culture surface, and six of these cultures showed an average of 5.9 beating muscle clusters: in total, 47 beating clusters were formed from eight EB cultures (Fig. 6C).

To test whether differentiation had occurred, quantitative PCR (qPCR) analyses were performed using harvested eight EB cultures. The goal of the qPCR was to quantify the expression of a pluri-potency marker (Pou5f1/Oct-4) that is a signal of undifferentiated ES cells was to quantify the occurrence of five three-germ-layer markers: Brachyury and Hand1 which are expressed in mesoderm; Gata4, which expressed in endoderm; and Sox1 and Otx2 which are expressed in ectoderm. Compared to undifferentiated ES cells, the ES cells that had been induced to differentiate using the ATPS method showed decreased mRNA expression of Pou5f1/Oct-4, highly elevated mRNA expressions of mesoderm lineage markers, and moderately increased mRNA expressions of endoderm and ectoderm lineage markers (Fig. 6D). The relative expression levels of the three germ layers were well corresponded with the beating cluster counting result because cardiac muscle originates from mesoderm. Moreover, gene expression profile of the cells differentiated using ATPS were almost the same as that of the cells differentiated using conventional HD method; this similarity implies that the ATPS method does not noticeably affect the differentiation capacity of embryonic stem cells (Fig. 6D).

## Discussion

Patterned cell cultures that use ATPS have been reported in several papers, but they focused only on the concentrations of phase-forming polymers; the studies overlooked the densities of phases, which can also substantially affect the behavior of the cells in the system. In this study, we reinterpreted the concept of polymeric concentration in ATPS as a density of phases, and developed a new method to induce formation of cell spheroids by adjusting the densities of ATPSs. The principles of cell flotation and spheroid formation in ATPS could be explained using simple force interactions (Fig. 2). This explanation indicates that the principle of the ATPS method is almost the same as the principle of the HD method, in which the cells are trapped at the interface between air and the culture medium, and gather at the drop apex due to gravitational force. Because of ATPS and HD exploit similar principles, the ATPS method would have capability form various types of spheroids such as random co-culture spheroids that is similar to the capability of HD. However, the capability of the ATPS method to form more-complex spheroids such as cell-in-cell or Janus spheroids, which are difficult to be generated by the HD method, was not tested.

However, the ATPS spheroid method has advantages over the HD method. First, compared to the HD method, the ATPS method is less affected by droplet evaporation and, thus the minimum size of droplet is not limited. For example, in this study, we used 0.5-μl DEX droplets to form spheroids, whereas the HD method uses >10 μl droplets. This use of small droplets in the ATPS method increases the chance of cell interactions even at low cell concentrations, and also provides a high meniscus curvature, which could facilitate uniform formation of a single spheroid in a droplet. For example, when 5,000-cell spheroids form, cells formed using the HD method (in 20-μl drop) about 40 times farther apart than those formed using the ATPS method (in 0.5-μl drop). As a proof of this, ATPS method showed more efficient (more spherical and tighter) spheroid formation than did the HD method (Fig. 4). Second, compared to the HD method, the ATPS method is more suitable for high-throughput applications due to its compatibility with conventional multi-well plates, and is therefore also compatible with existing automated liquid handlers. Moreover, the ATPS method can confine spheroid attachment to a limited DEX area because the diluted ATPS pattern still has enough interfacial tension to keeps the spheroids from escaping, so they are always attached near the center of the culture surface; this positioning guarantees maximized spreading area.

Although some techniques such as commercialized HD plates and transfer plates can also provide efficient and easy spheroid manipulations, none of them can be used for the direct seeding of spheroids onto culture surface without transferring spheroids and changing vessels. For example, commercially available GravityPLUS/TRAP (InSphero) and HD Plates (BioMatrix) can provide efficient and convenient HD and spheroid transfer platforms for suspension cultures, but do not provide any platforms for spheroid seeding and adhesion cultures which are required in some experiments such as spheroid spreading (or invasion) assays and monolayer-spheroid co-cultures. In addition, high-throughput spheroid formation systems that use non-adhesive surfaces or structures such as Aggrewell (Stem Cell Technologies) are also commercially available for some applications that require bulk generation of spheroids, but the systems are generally less suitable for single spheroid analyses than is ATPS.

The most important attribute of the ATPS method is its ability to switch culture mode from floating to adhesion culture. Because the method can form, release and attach spheroids in the same wells, the probability of spheroid loss, breakage and merger during spheroid manipulation is minimized; therefore the reliability and consistency of results could be maximized. Because this whole procedure for spheroids adhesion culture can be achieved by only a single pipetting in ATPS method, there are no other methods that can achieve single spheroids/well adhesion culture easier than ATPS method does. Although this method may not be compatible with cells such as HepG2 that form spheroids slowly, our mES cell differentiation example indicates that PEG/DEX ATPS is compatible with most cell lines, including very sensitive stem cells. Therefore, these unique characteristics of the ATPS spheroid-forming method make it applicable to various areas of spheroid research, such as spheroid-surface or spheroid-cell monolayer interactions^22^,^23^.

In summary, we developed a new method to culture cell spheroids that uses a density-adjusted PEG/DEX aqueous two-phase system. This simple method can generate various size-controlled spheroids in a conventional multi-well plate without complex procedures. Moreover, because the formation of spheroids in the ATPS pattern is mainly affected by the density of the phases, the culture mode can be switched easily from floating spheroid culture to surface-attached adhesion culture by adding a few drops of polymer-free medium. This simple switching can reduce the number of laborious spheroid manipulation steps such as spheroid collection, transfer and distribution, and can therefore also reduce the errors caused from those manipulations. Using this method, we successfully induced differentiation of mouse embryonic stem cells into beating cardiac muscle cells without any laborious spheroid manipulation procedures. Due to its technical simplicity and compatibility with conventional culture platforms, this method can be combined with automatic liquid handlers to achieve a high-throughput system for spheroid formation and analysis.

## Materials and Methods

### Preparation and density measurement of PEG/DEX ATPSs

The phase diagram of 35 K Da PEG (Sigma Aldrich) and 500 K Da DEX (Pharmacosmos) was plotted (Fig. 1A) to determine the threshold concentration for phase separation as previously described^24^. Based on this diagram, eight concentrations (wt%/wt%) of PEG/DEX ATPSs (5/1, 5/3, 5/5, 5/7, 5/9, 5/11, 5/13 and 5/15) were selected and prepared by dissolving appropriate amounts of PEG and DEX in 30 ml complete growth cell culture media. To completely dissolve these media with added polymer, they were incubated on a rocking machine for >8 h at 4 °C in a cold room or refrigerator. Completely dissolved PEG/DEX media were centrifuged at 3000 g for 15 min at 4 °C to separate the phases. The separated PEG-rich phase (top phase) and DEX-rich phase (bottom phase) were transferred to new containers and kept at 4 °C until use. The density of prepared PEG- and DEX-rich phases from the eight ATPSs were calculated from precisely-measured weights and volumes using an electronic balance and volumetric flasks (WITEG).

### Preparation of DEX-in-PEG ATPS patterns

To generate DEX-in-PEG ATPS patterns, pre-separated PEG-rich and DEX-rich phases were first centrifuged again at 16000 g for 10 min at 4 °C to remove unseparated or undissolved traces of DEX and PEG. Then the appropriate quantity of PEG-rich phase (100 μl for 96-well plate) was transferred to a culture vessel and 0.5 μl of DEX-rich phase was dotted inside the PEG-rich phase using micro-pipettes (Fig. 1B). To avoid bubble generation, all liquids were carefully pipetted and transferred.

### Cell culture

Four human tumor cell lines (MCF-7, HepG2, HCT 116 and MDA-MB-231), one mouse embryonic fibroblast cell line (NIH-3T3) and one mouse embryonic stem cell line (ES D3) were used in this study. MCF-7 and HepG2 cells were purchased from the Korean Cell Line Bank (KCLB) and maintained using minimum essential medium containing eagle’s salts (MEM, Gibco) supplemented with 10% fetal bovine serum (FBS, Hyclone) and 1% antibiotics (P-S, Gibco). HCT116 human colon tumor cells and MDA-MB-231 cells were also purchased from KCLB and maintained using RPMI (Gibco) supplemented with 10% FBS and 1% antibiotics. NIH-3T3 cells were purchased from American Type Culture Collection (ATCC) and maintained using Dulbecco’s modified Eagle medium (DMEM, Hyclone) supplemented with 10% FBS and 1% antibiotics. Mouse embryonic stem cells (ES D3, ATCC) was purchased from ATCC. ES cells were seeded on 0.2% gelatin (Sigma Aldrich) coated dishes and maintained using knockout DMEM (KO DMEM, Gibco) supplemented with 15% knockout serum replacement (KOSR, Gibco), 1% antibiotics, 4 mM L-glutamin (Gibco), 0.1 mM 2-mercaptoethanol (Sigma Aldrich) and 10 ng/ml leukemia inhibitory factor (LIF, ORF). In differentiation experiments, Iscove’s modified Dulbecco’s medium (IMDM, Gibco) supplemented with 20% FBS and 1% antibiotics was used instead of ES complete growth medium.

### Density measurement of cells using gradient centrifugation

Densities of the cells were measured using discontinuous Percoll density gradient centrifugation. First, 100% working solution was prepared by mixing 1 part of 10X PBS and 9 parts of Percoll solution (Sigma Aldrich) to adjust osmolality of the density gradient medium. This 100% working solution was then further diluted to 80, 60, 40 and 20% with appropriate volumes of PBS or of PBS containing 0.1% phenol red (Sigma Aldrich), which helps to visualize the layers of the gradient medium. Various concentrations of prepared gradient media were layered in 15-ml conical tubes, then 5 × 10^6^ cells in PBS were layered on top of them. These tubes were then centrifuged at 400 g for 20 min at room temperature (RT) and the position of the cells was recorded. The densities of various concentrations of Percoll solutions were calculated using a formula in the manufacturer’s instructions.

### Cytotoxicity determination using live/dead staining and trypan blue exclusion assay

Cells were first seeded in 24 well tissue culture plate and cultured for 24 h. Media of seeded cells were then changed to PEG- and DEX-rich phases of 5%/9% ATPS and cultured for 48 h to determine the cytotoxicity of the ATPS. The PEG- and DEX- rich phase treated cells were stained using calcein AM and ethidium homo-dimer to determine viability. Viability of cells were calculated according to manufacturer’s instruction. In the case of embryonic stem cells, cells were detached from plate using trypsin and counted with trypan blue to determine viability because the cells grow as dense colonies.

### Spheroid generation using density adjusted DEX-in-PEG pattern ATPS

Cells were harvested as 2 × 10^6^ cells/ml suspensions, and desired numbers of cells where transferred to 1.5-ml micro-centrifugation tubes and pelleted at 200 g for 3 min at RT. These pellets were resuspended in 200 μl DEX-rich phases as a homogeneous single cell suspension, then 0.5 μl of these DEX-rich phases containing cells were patterned in PEG-rich phases (section 2.1.2). Generally, 1 × 10^6^ cells/0.2 ml for MCF-7, HCT 116 and NIH-3T3, 2 × 10^6^ cells/0.2 ml for ES D3 and 5 × 10^5^ cells/0.2 ml for HepG2 cells were used to form patterns. In the size-control test, 2 × 10^6^, 1 × 10^6^, 5 × 10^5^, 2.5 × 10^5^, 1.25 × 10^5^ and 0.63 × 10^5^ cells/0.2 ml HCT 116 cells were used. All spheroid generation experiments were performed in 5%/9% PEG/DEX ATPSs after PEG/DEX concentration-dependency of spheroid formation was confirmed using six ATPS concentrations (wt%/wt%) (5/3, 5/5, 5/7, 5/9 and 5/11 PEG/DEX).

### Spheroid formation using hanging drop

Spheroids were formed using well known hanging drop method. Harvested cells were suspended in growth media at the concentration of 5,000 cells/20 μl. Drops of prepared cell solution (20 μl/ drop) were then patterned on a lid of 60-mm tissue culture plate and incubated for 2 days to form spheroids.

### Measurement of spheroid size

Spheroids formed with HCT 116 cells were imaged using an Olympus IX71 Inverted microscope, and the images were analyzed using ImageJ software (NIH). Briefly, all images were converted to simplified threshold images under the same converting condition and the edges of the spheroids were then detected using a selection tool. Feret’s diameters of the detected spheroid edges were measured initially as pixels, and converted to micrometers by comparing to a reference length.

### Release and attachment of formed spheroid

Forty-eight hours after ATPS patterning, 200 μl of PEG- and DEX-free fresh media were carefully added to the wells containing spheroids without disrupting the ATPS patterns. Twenty-four hours after the addition of fresh media, spheroids were observed under a microscope to determine whether they had released from the apex and attached to surface. After confirming spheroid attachment to the culture plate surface, all liquids were removed from the wells and 200 μl fresh media were added for further spheroid cultivation and analysis.

### ES cell differentiation using ATPS

To differentiate ES cells, DEX-rich phase containing 2 × 10^6^ ES D3 cells/0.2 ml was patterned on a 96-well plate with DEX-in-PEG ATPS patterns made with ES cell growth medium. Forty-eight hours after patterning, the floated embryoid bodies (EBs) were released and patterned on the culture surface by adding fresh differentiation medium. This day was defined as day 0 for the experiment. On day 1, media containing polymer were refreshed with two PBS washings after confirming EB attachment; the media were then refreshed every three days until day 9. On day 12, the numbers of beating clusters were counted under a microscope and cells were harvested for further analysis of three germ layer markers.

### Quantitative polymerase chain reaction (qPCR) analysis

On day 12, RNA of the harvested cells was extracted using TRI reagent (Sigma Aldrich) and chloroform (Sigma Aldrich) following the manufacturer’s instructions. The isolated RNAs were then precipitated using isopropyl alcohol (Sigma Aldrich) and washed using ice-cold ethanol (Sigma Aldrich). Prepared RNAs were then quantified using a spectrophotometer (Jenway, Genova) and converted to cDNA using reverse transcriptase (RT-PCR, Promega). To measure relative expression levels of representative three germ layer markers, prepared cDNAs were analyzed using a One Step SYBR green quantitative PCR kit (TaKaRa Bio) and Light cycler 2.0 (Roche) using specific PCR primers. (Table S1) Measured mRNA expressions of three germ layer markers were normalized by actin expression and further divided by the normalized expression levels from undifferentiated ES cells.

## Additional Information

**How to cite this article**: Han, C. *et al.* Formation and manipulation of cell spheroids using a density adjusted PEG/DEX aqueous two phase system. *Sci. Rep.* **5**, 11891; doi: 10.1038/srep11891 (2015).

## Supplementary Material

Supplementary Information

Supplementary Information
