# Supplementary material for: Formation and manipulation of cell spheroids using a density adjusted PEG/DEX aqueous two phase system
Source: Sci Rep. 2015 Jul 6;5:11891. doi: 10.1038/srep11891 (PMC4491721; doi:10.1038/srep11891)
Supplement: Supplementary Information [file srep11891-s2.pdf]

**Supplementary Information**

# Formation and manipulation of cell spheroids using a density adjusted PEG/DEX aqueous two phase system

Chungmin Han[1], Shuichi Takayama[2*] and Jaesung Park[1,3**]

**Figure S1**

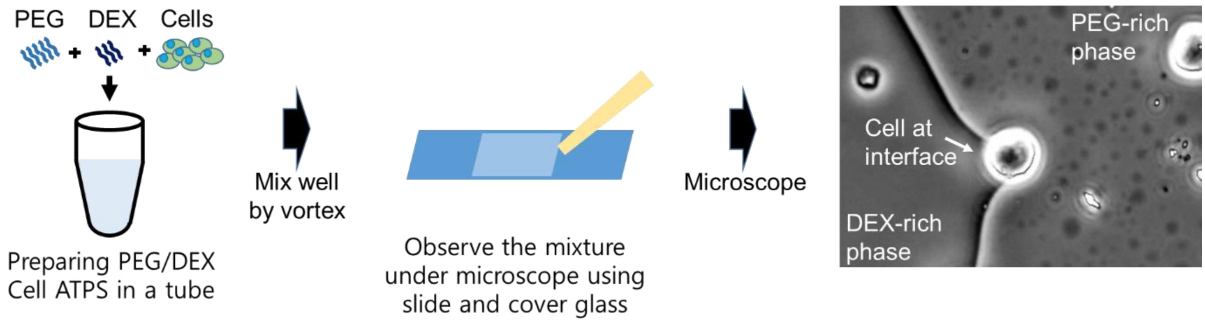


Contact angle measurement. Well-mixed PEG/DEX/cell mixture was observed under phase-contrast microscope. Sample was mount using slide and cover glasses. By chance, a few cells were trapped at PEG/DEX phase interface. Contact angle observation indicated that DEX-cell interactions are more favorable than PEG-cell interactions.

**Figure S2**

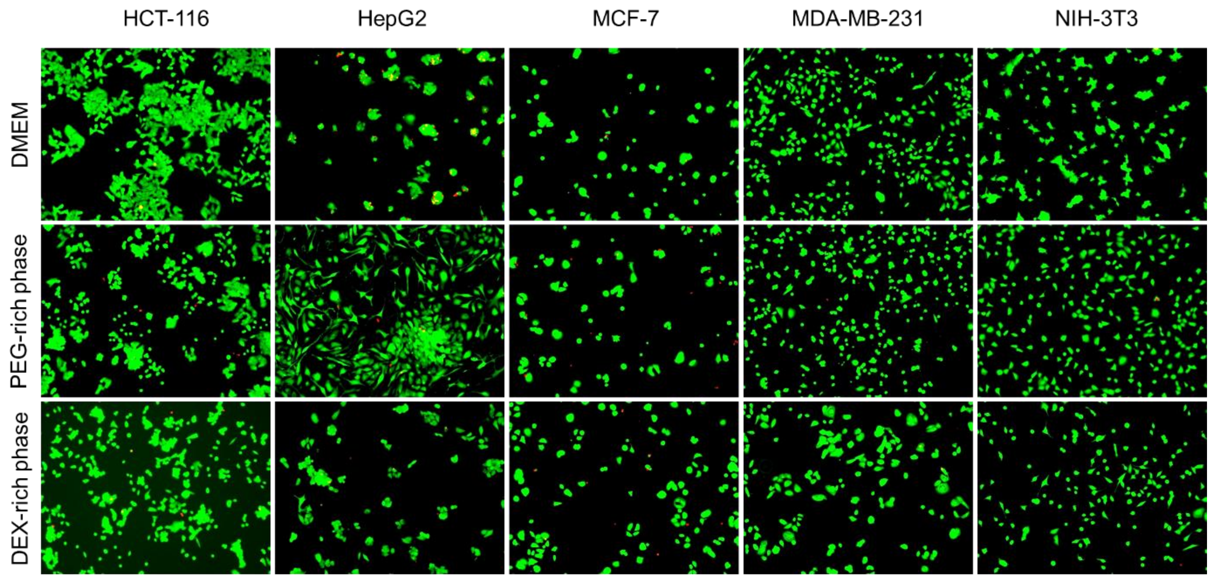


PEG and DEX cytotoxicity determination. HCT-116, HepG2, MCF-7, MDA-MB-231 and NIH-3T3 cells were first cultured with normal growth media, PEG- and DEX-rich phases for 48 hours. After that, cells were stained with calcein AM and ethidium homo-dimer for live and dead analysis. Green (calcein AM) signal indicates live cells and red (ethidium homo-dimer) signal indicates dead cells.

**Figure S3**

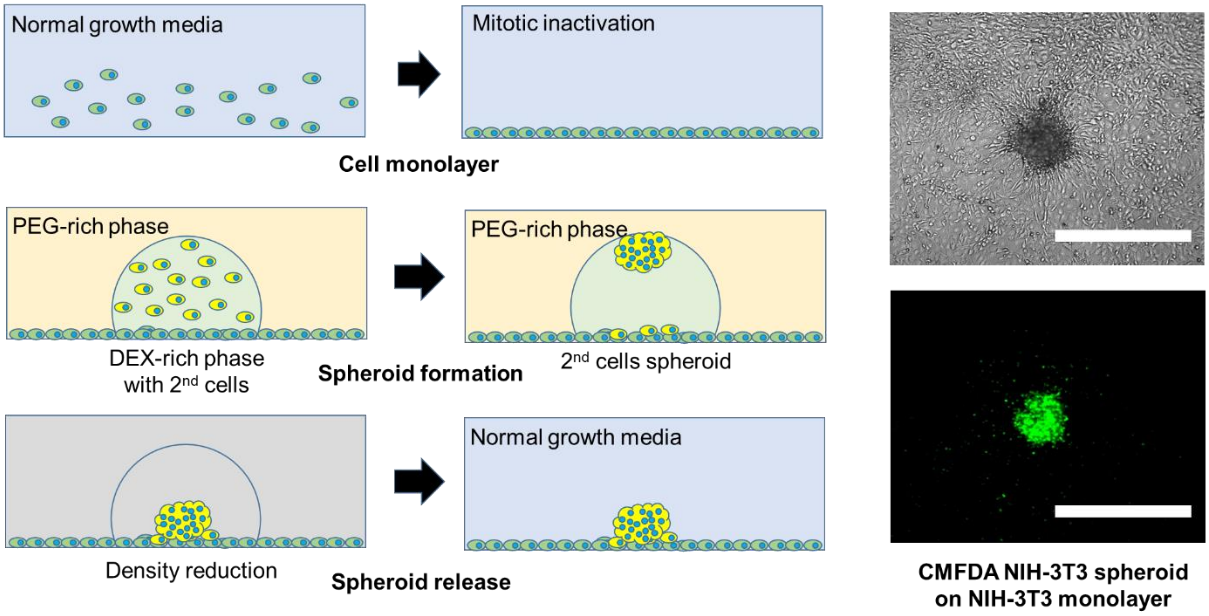


Spheroid-monolayer co-culture system. First, 1st cell types were culture as monolayer in normal growth media. After the culture reaches ~90% confluent, cells were mitotically inactivated by mitomycin c treatment. (left top) Then, 2nd cell types were patterned using DEX-in-PEG ATPS for spheroid formation for 2 days. (left mid) When spheroid was formed, polymer free media was added to the culture to release the spheroid on previously formed 1st cell type monolayer. After 1 day incubation, polymer media was changed to fresh and polymer free media for further analysis. (left bottom) Microscopic picture was taken with CMFDA labeled NIH-3T3 spheroid cultured on non-labeled NIH-3T3 monolayer. (right) Scale bars: 400 μm

**Table 1S**. Primers for real time quantitative PCR

| Name | Ref. Seq. # | Sequence | Size (bp) | Etc. |
|---|---|---|---|---|
| Actb (Actin) | NM_007393.3 | Fwd- ACGTTGACATCCGTAAAGAC | 100 | House Keeping |
| | | Rev- GCAGTAATCTCCTTCTGCAT | | |
| Pou5f1 (Oct-3/4) | NM_013633.3 | Fwd- GTTGGAGAAGGTGGAACCAA | 61 | Pluripotency |
| | | Rev- CTCCTTCTGCAGGGCTTTC | | |
| Brachyury | NM_009309.2 | Fwd- CAGCCCACCTACTGGCTCTA | 72 | Mesoderm |
| | | Rev- GAGCCTGGGGTGATGGTA | | |
| Hand1 | NM_008213.2 | Fwd- CAAGCGGAAAAGGGAGTTG | 84 | Mesoderm |
| | | Rev- GTGCGCCCTTTAATCCTCTT | | |
| Gata4 | NM_008092.3 | Fwd- GGAAGACACCCCAATCTCG | 72 | Endoderm |
| | | Rev- CATGGCCCCACAATTGAC | | |
| Sox1 | NM_009233.3 | Fwd- GTGACATCTGCCCCCATC | 60 | Ectoderm |
| | | Rev- GAGGCCAGTCTGGTGTCAG | | |
| Otx2 | NM_144841.4 | Fwd- AGAATCCAGGGTGCAGGTAT | 98 | Ectoderm |
| | | Rev- CAGGCCTCACTTTGTTCTGA | | |
